# Improving rural doctors’ professional competence: effectiveness analysis of China’s village doctors policy

**DOI:** 10.3389/fpubh.2025.1591633

**Published:** 2025-05-07

**Authors:** Jiantao Li, Tinghui Lian, Yayan Tian, Yuxiao Wang, Jingru Zhang, Meichen Wu

**Affiliations:** Department of Health Economics, School of Management, Shanxi Medical University, Taiyuan, China

**Keywords:** village doctor, professionalization, village doctor policy, propensity score matching (PSM), township-hired village doctors

## Abstract

**Objectives:**

This study aimed to analyze the impact of the Village Doctor Policy on the professionalization of village doctors.

**Methods:**

Data were collected from a field survey conducted in Western China in 2023. The Professionalism Level Measurement Questionnaire was constructed using exploratory factor analysis (EFA) and confirmatory factor analysis (CFA) to ensure its validity. Propensity score matching (PSM) analysis was employed to compare the professionalization levels of village doctors in areas with and without policy implementation.

**Results:**

The sample had more males than females, with most participants aged 46–60 years (48.4%). Ordinary least squares (OLS) regression indicated that the policy positively influenced the professionalization of village doctors (*p* < 0.001), statistically significant effects on professional skills (*β* = 7.02, *p* < 0.01), professional conduct (*β* = 5.89, *p* < 0.05), and professional awareness (*β* = 6.34, *p* < 0.05). To address potential selection bias, PSM was conducted using three matching techniques. The average treatment effect on the treated (ATT) was 6.536 for k-nearest neighbor matching, 9.017 for caliper matching and 6.110 for kernel matching. These findings further confirmed the positive impact of the policy. The enhancement of professional skills, conduct, and awareness was found to be statistically significant (*p* < 0.05) under caliper matching.

**Conclusion:**

The Village Doctor Policy enhanced the professionalization of village doctors and strengthened Village doctor corps. This policy transformed the “half doctor, half farmer” status of village doctors to “unit members,” promoting their professional development and improving medical service quality. It is recommended that the government continue to promote localized rural employment policies to enhance the motivation and incentives of health care professionals.

## Introduction

Universal health coverage is one of the global sustainable development goals (1), and primary healthcare is essential to achieving it (2). Primary healthcare workers have been characterized as the backbone of the entire health system, linking communities, health systems and other sectors (1). In China, village doctors have always been an important part of the medical and health workforce, guardians of rural residents’ health, and the cornerstone of primary health care (3).

Rural doctors appeared in China in the 1950s, initially part-time by local farmers who received basic medical and nursing training, also known as “barefoot doctors” (4). Since the 1980s, this model has evolved into a more professionalized system, with village doctors now responsible for essential public health services such as health record maintenance, health education, infectious disease control, and chronic disease management (5). Evidence from rural China demonstrated that comprehensive hypertension interventions led by village doctors significantly improved blood pressure control rates and validated their irreplaceability in terms of patient education, long-term follow-up, and cost control (6).

However, with economic development, population mobility and increased urbanization, the development of village doctors faces many challenges. A 2021 study showed that 70% of China’s village doctors are over the age of 40 (7). Rural areas possessed 2.74 physicians plus assistant physicians (AP) per 1,000 population in 2024, less than the rate of urban regions which reached up to 4.13 in the same year (8). This reflects the aging and staffing shortages faced by village doctors. Relevant studies also confirmed that economic dissatisfaction, concerns about occupational environment and organizational management are important factors affecting the resignation intention of village doctors (9). Notably, a study revealed that only 44.2 percent of village doctors were capable of fully and accurately managing common illnesses (10). This shows that the current service capacity of rural doctors is insufficient to meet patient demand. Insufficient career attraction (low income and poor working conditions), high brain drain, serious aging, imperfect training system, and insufficient policy support and resource allocation are the main problems currently facing the development of village doctors in China (11).

In order to improve the stability and service capacity of the rural doctor workforce, the Chinese government began to promote the village doctor policy in 2021. The “Township-Hired Village Doctors” is the core element of China’s village doctor policy (12). “Township-Hired Village Doctors” refers to the recruitment of village doctors by township health centers (or county-level health departments) on an employment contract basis and their posting to work in village clinics, so that they can be integrated into the unified management of primary medical and healthcare institutions and enjoy basic salaries, performance subsidies and social security, in order to Enhance the overall quality of the rural doctor workforce. By signing employment contracts with township health centers, the career attractiveness and stability of village doctors has been significantly enhanced (13). Shanxi Province’s “Township-Hired Village Doctors” policy, which is due to be officially implemented in 2021, is the first province to incorporate the treatment and pension protection of village doctors into its legal system, and stipulates that eligible village doctors may sign contracts and participate in basic pension insurance for employees. In this background, it is interesting to analyze the impact of “Township-Hired Village Doctors” on the service capacity of the village doctor workforce.

The theory of professionalization provides an important theoretical perspective for assessing the impact of Chinese rural doctors’ policies on their professional competence. According to existing research, professionalization refers to the standardization of occupational roles, the construction of knowledge-based practices, and the promotion of institutional legitimacy and autonomy of the occupation. Guo Y. pointed out that professionalization can be analyzed from a number of dimensions such as occupational knowledge systems, normative frameworks, organizational safeguards, and social identity (14). And Li H. emphasizes that the essence of professionalization lies in the transformation of informal or semi-professional roles, into formal occupations dominated by professional norms (15).

This study analyzes village doctors’ professionalization in terms of competence, institutional identity and service quality under policy intervention using professionalization theory. It employs propensity score matching (PSM) to assess the “Township-Hired Village Doctors” and its impact on professionalization. By evaluating professionalism through a questionnaire, this study provides evidence-based insights and policy recommendations for enhancing China’s rural healthcare workforce development.

## Methods

### Study design and participants

The study adopted a cross-sectional survey design using multi-stage cluster random sampling method in which two districts were selected from each of the implementation and non-implementation areas. In these counties, 4–6 townships were randomly selected for the questionnaire survey of rural doctors. A total of 363 questionnaires were distributed, achieving a 100% response rate. After excluding 4 invalid questionnaires (containing over 10% missing items, exhibiting inconsistencies, or incomplete responses), 359 valid questionnaires were retained for analysis, yielding a valid response rate of 98.44%.

The team of rural doctors in Shanxi Province faces the outstanding contradiction of ‘one high and three lows’ (high age, low education, low qualification and low income), which is highly similar to the predicament that exists in rural areas across the country and is of general research significance. Shanxi Province has diverse geographic characteristics, covering areas with different levels of development, such as economically underdeveloped, urban–rural integration belts, and densely populated areas. At the same time, Shanxi Province is an ideal sample to study the development of village doctors’ professionalization level by virtue of its policy innovation, typicality, geographical diversity and data accessibility.

### Questionnaire design

The questionnaire consists of three parts: theme and introduction, demographic information and professionalization level. Professionalization refers to an occupational field defined by specialized knowledge, authoritative expertise, and ethical standards, highlighting the necessary skills and qualities of practitioners (16).Current research on professionalization mainly addresses counselors, construction workers, and corporate employees. Studies of village doctors often focus on issues such as functional roles, working methods, entry and exit protocols, professional treatment, and pension security. Direct research on the outcomes of village doctors’ professionalization is limited. Therefore, by referencing prior studies that outline the professionalization mechanisms for village doctors (17), we identified commonly used dimensions and indicators for evaluating professionalization levels. Combining these with the evaluation standards from the “Village Clinic Service Capability Standards (2022 edition),” we initially developed an evaluation system for village doctors’ professionalization levels, generating a total of 29 items.

The study starts from three dimensions of professional awareness, professional behavior, and professional skills: professional awareness (VA1-VA17) measures the extent to which rural doctors identify with their own professional identity, ethical responsibilities, and social values, covering a sense of work mission (such as “I believe that the work of village doctors is crucial to the health of villagers”) (18); professional behavior (VB1-VB6) mainly refers to whether practitioners comply with language, behavioral norms, and image standards of their professional identity (such as “whether they wear white coats while on duty”) (19); professional skills (VC1-VC6) mainly refer to professional knowledge updates and practical abilities, such as skills in managing chronic diseases (such as “ability to follow up on high-risk groups with chronic diseases”) (20). The main content covers professional skills, conducts and awareness, with items numbered VA1 to VA17, VB1 to VB6, and VC1 to VC6. A 5-point Likert scale was used, where “1” to “5” denote “strongly disagree” to “strongly agree.” Additionally, five health policy scholars (including two professors), three county-level health commission administrators, and two senior rural doctors were invited to form a consulting group to provide modification suggestions regarding dimension division and item descriptions. The survey, including the pre-survey and formal survey, was conducted at village clinics in the Shanxi Province. The rural doctor team in Shanxi Province faces the prominent contradiction of “one high and three lows” (high age, low educational background, low practice qualifications, low income), which is highly similar to the common dilemma in rural areas nationwide, making it of universal research significance. Shanxi Province has diverse geographical characteristics, covering regions with different levels of development such as underdeveloped economy, urban–rural integration zones, and densely populated areas. With its policy innovation, typical problems, geographical diversity, and data accessibility, Shanxi Province is an ideal sample for studying the development of the professionalization level of rural doctors. The village doctors participated and four trained team members distributed the questionnaires.

### Propensity score matching and variable specification

To investigate the relationship between village doctors’ professionalization levels and the Village Doctor Policy, the following statistical methods were employed: t-tests were conducted to assess professionalization level differences between policy-implemented and non-implemented areas. Given that the dependent variable was continuous, an Ordinary Least Squares (OLS) regression model was used to predict this relationship (Model 1). In Model 1, the professionalism level_i_ represents the professionalization level of the village doctor: TRVU represents whether the Village Doctor Policy is implemented in the area where the i-th Village doctor is located; X_2_…X_k_ represents other control variables; β _0_ is the intercept term; β _1_ is the correlation coefficient matrix, indicating how TRVU affects the professionalization level of village doctors; β _2_… β _k_ are the correlation coefficients of how other control variables affect the professionalization level of village doctors, and ε _i_ is the random error term. Propensity Score Matching (PSM) was employed to estimate the policy’s impact, addressing potential endogeneity arising from sample self-selection bias. The Village Doctor Policy is influenced by factors such as gender and education level, leading to non-randomness and potential endogeneity issues from sample self-selection bias, which could affect model estimations. The PSM model can theoretically mitigate confounding factors that cause selection bias and address potential endogeneity issues (21). The theoretical framework of matching estimation is the counterfactual reasoning model, which identifies control group samples that are similar to those in the treatment group to match counterfactual individuals. Thus, the PSM model helps to reduce bias and ensures estimation consistency. PSM involves four steps: calculating the propensity score using a logit model, matching based on this score, assessing the balance after matching, and calculating the average treatment effect (ATT). This study used nearest-neighbor matching, radius matching, and kernel matching. Samples were categorized into two groups: the Village Doctor Policy Implementation group (treatment group) and the non-implementation group (control group). Model 2 specifically demonstrated the impact of the average treatment effect (ATT) on the professionalization level of village doctors. Y_1_ represents the average value of the explanatory variables when samples from the treatment group received the intervention, whereas Y_0_ represents the average value assuming that they did not receive the intervention.


$${\mathrm{Professionalism level}}_{i}=+\beta_{1}\mathrm{TRVU}+\beta_{2}X_{i1}+\cdots +\beta_{k}X_{ik}+\varepsilon_{i}\;\left(\mathrm{model}\;1\right)$$

$$ATT=E\left(Y_{1}|p=1\right)-E\left(Y_{0}|p=1\right)\;\left(\mathrm{model}\;2\right)$$

#### Control variables

Control variables were selected based on previous studies (22–25), including age, sex, academic background, professional level and title, annual work income, average daily working hours, educational background, and years of work experience.

#### Independent variable

The implementation of the Village Doctor Policy was assessed utilizing a dichotomous variable (“yes” or “no”), with verification conducted by local health administrative personnel and officials to confirm the policy’s enforcement.

#### Dependent variable

The professionalization level of village doctors was evaluated using a self-administered questionnaire. This scale was subjected to exploratory and confirmatory factor analyses, which yielded favorable outcomes.

### Data collection procedures

The questionnaire was administered between August 2023 and October 2023 through offline field research. Participants were rural doctors working in village clinic. Informed consent was obtained prior to participation in the survey to ensure ethical compliance.

### Statistical analyses

The validity and reliability of the questionnaire were assessed using SPSS 26.0 and AMOS 26.0 through item analysis, exploratory factor analysis (EFA), and confirmatory factor analysis (CFA). In the pre-test phase, data from a subsample (*n* = 105) were used to conduct item discrimination analysis using the extreme group method, as well as reliability testing via Cronbach’s alpha. EFA was employed to explore the underlying structure of the professionalization construct, identifying three distinct dimensions: professional skills, professional conduct, and professional awareness. Items were reduced based on exploratory factor analysis (EFA) using these criteria: (1) measure loaded less than 0.4 on all common factors; (2) measure loaded greater than 0.4 on two or more common factors; and (3) indicator was categorized differently than expected and could not be reasonably interpreted.

Subsequently, CFA was conducted on the full sample (*n* = 359) to validate the three-factor structure and test the model’s structural validity. Assessment criteria include: standardized factor loadings between 0.5 and 0.95; standard errors (SE) < 1 and positive; critical ratios (CR) > 2.58; and average variance extracted (AVE) > 0.5. Convergent validity was confirmed by composite reliability (CR > 0.6) and AVE (≥ 0.5). For discriminant validity, the square root of AVE must exceed standardized correlation coefficients between variables. Model fit is reflected by absolute, incremental, and parsimonious indices. The absolute fit indices included the RMSEA value (<0.08), GFI value (>0.9), and AGFI value (>0.9); the incremental fit indices include the IFI value, TLI value, and CFI value, all of which should be greater than 0.9; the parsimonious fit index, $\chi^{2}/df$ , should be less than 5. Common method bias was examined using the latent variable control method by comparing model fit between a baseline model A0 and a method-factor model (A1). Significant improvement in A1’s fit indices (e.g., RMSEA, CFI, GFI, AGFI, TLI) would indicate common method bias.

To assess the influence of policy implementation while addressing selection bias, propensity score matching (PSM) was conducted using Stata 17. Logit models were used to predict propensity scores based on their level of professionalism for analysis. At the same time, we conducted common support domain assessment and equilibrium tests to verify the robustness of the propensity score matching method.

## Results

### Descriptive statistics

The sample had more males than females, with most participants aged 46–60 years (48.4%). Many had over 20 years of experience as village doctors and 41.75% earned less than 30,000 RMB annually. Education levels were primarily high school or vocational diplomas. Table 1 details the demographic variables, where N_0_ and N represent the total number of pre-survey and survey samples, respectively, and n_0_ and n denote the corresponding example counts for the different categorical characteristics. The composition ratio shows the proportion of each categorical characteristic count relative to the total count expressed as a percentage.

**Table 1:** Participants characteristics.

Variables	Sample (n_0_/n)	Percentage (%)
Gender		
Male	61/205	58.1/57.1
Female	44/154	41.9/42.9
Age		
≤30	17/44	16.2/12.3
31–45	33/126	31.4/35.1
46–60	52/174	49.5/48.4
>60	3/15	2.9/4.2
Years of service as a village doctor		
≤10	33/107	31.4/29.8
11–20	24/81	20/22.6
21–30	34/109	32.4/30.3
>30	17/62	16.2/17.3
Annual working income (Yuan)		
≤ 20,000	39/132	37.1/36.8
20,000–30,000	38/115	35.3/32
30,000–40,000	18/80	17.1/22.3
>40,000	11/32	10.5/8.9
Education		
Primary school or below	0/1	0/0.3
Junior school	5/7	4.8/1.9
High school or vocational school	51/205	48.6/57.1
Associate degree	49/144	46.7/40.1
Undergraduate and above	0/2	0/0.6

N_0_ = 105, N = 359. n_0_ represents the characteristics of our pre-survey sample size, n representing the total sample size.

### Questionnaire validation

#### Item analysis

As shown in Table 2, the preliminary survey data results indicate that the *p*-values (two-tailed) for all items are less than the standard significance level of 0.05, which meets the level of significance and suggests that each item has good discriminating power. Subsequently, correlation coefficient analyses showed that the corrected item-total correlation coefficients (CITC) for all items met the established criteria, with an overall reliability coefficient of 0.961.

**Table 2:** Preliminary survey analysis results.

Dimension	Indicator	Sig(2-tailed)	CITC	Dimensionally cronbach’s *α* coefficient	Cronbach’s *α* coefficient
Professional skill	Your ability to recognize, diagnose, and treat common diseases (VA1)	<0.001	0.785	0.956	0.961
	The challenge in aiding the township health center or medical community to standardize clinic visits, rounds, and documentation of clinic visits (VA2)	<0.001	0.718		
	How difficult it is for you to manage the health of key populations and chronic disease patients with Chinese medicine and provide Chinese medicine health care services (VA3)	<0.001	0.746		
	Challenges in providing immediate testing for items like electrocardiograms, routine blood tests, and urine tests (VA4)	<0.001	0.691		
	Coverage rate of electronic health records for permanent residents in the jurisdiction (VA5)	<0.001	0.626		
	Number of health education activities conducted in cooperation with towns and village committees (VA6)	<0.001	0.712		
Professional skill	Challenges in identifying and reporting suspected adverse reactions after vaccination (VA7)	<0.001	0.701	0.956	0.961
	Challenges in neonatal visits, neonatal disease screening, and monitoring children’s growth and development (VA8)	<0.001	0.715		
	Your ability to effectively mobilize pregnant women for prenatal care (VA9)	<0.001	0.759		
	Your ability to provide health management services for individuals aged 65 and above (VA10)	<0.001	0.726		
	The difficulty in ensuring timely treatment or referral for older adult patients identified with diseases during health check-ups (VA11)	<0.001	0.781		
	Your ability to conduct health education, screening, and identification of patients with chronic diseases (VA12)	<0.001	0.766		
	Your ability to perform regular follow-ups with high-risk groups for chronic diseases (VA13)	<0.001	0.801		
Professional skill	Your ability to establish health management registries for the older adult and children aged 0–36 months, and provide traditional Chinese medicine healthcare services (VA14)	<0.001	0.780	0.956	0.961
	Your ability to register and report information on infectious disease outbreaks and public health emergencies (VA15)	<0.001	0.759		
	Your ability to conduct epidemiological investigations, follow-ups, and manage close contacts (VA16)	<0.001	0.698		
	You’re training and ability to offer consulting services and technical guidance on eugenics and family planning (VA17)	<0.001	0.749		
Professional conduct	You are able to prescribe according to the ‘Prescription Management Regulations’ and write prescriptions in a standardized manner (VB1)	<0.001	0.792	0.906	
	You can sort medical waste and store it separately from household waste with proper management registration (VB2)	<0.001	0.717		
	You can regularly inventory medications to ensure there are no expired drugs (VB3)	<0.001	0.716		
	You can use standardized professional terminology that does not affect the understanding of villagers during diagnosis and treatment (VB4)	<0.001	0.700		
	You can always wear a white coat while on duty (VB5)	<0.001	0.737		
	You can refrain from using personal communication devices during diagnosis and treatment (VB6)	<0.001	0.818		
Professional awareness	You have your own work goals and can strive to achieve them (VC1)	<0.001	0.756	0.891	0.961
	You have a clear understanding of your position and job responsibilities (VC2)	<0.001	0.704		
	You can work well with other medical institutions (VC3)	<0.001	0.649		
	You are proactive in receiving medical education (VC4)	<0.001	0.730		
	You are proactive in improving your diagnostic and treatment skills (VC5)	<0.001	0.771		
	You possess a public service consciousness dedicated to serving and contributing to the community (VC6)	<0.001	0.644		

#### Reliability analysis

The results shown in Table 2 indicate that the overall Cronbach’s alpha coefficient of the indicator system is 0.961, which is considered highly reliable. The reliability of each subscale ranged from 0.891 to 0.956, which is acceptable. This also suggests that the internal consistency of the indicator system designed in this study is very good.

#### Exploratory factor analysis

For a sample size of 105, the KMO value was 0.938, which exceeded the threshold of 0.7. The Bartlett’s test yielded a chi-square value of 2175.444 with 406 degrees of freedom and a significance level of less than 0.001, which indicated that it was suitable for factor analysis. The Kaiser criterion extracted the factors with eigenvalues greater than 1 by principal component analysis followed by maximum variance rotation. Only factor loadings above 0.4 were displayed and ranked. The rotation converged after five iterations and extracted three factors with a cumulative variance contribution of 63.534%. The factor structure was consistent with the pre-determined structure and the results of factor loadings and cumulative variance contribution are shown in Table 3.

**Table 3:** Results of exploratory factor analysis of pre-survey data.

Variables	Factor loading			Factor (cumulative variance contribution rate)
	Factor F1	Factor F2	Factor F3	
VA11	0.776			32.194%
VA15	0.766			
VA17	0.759			
VA9	0.756			
VA6	0.738			
VA13	0.734			
VA1	0.732			
VA14	0.719			
VA2	0.716			
VA4	0.716			
VA8	0.691			
VA7	0.689			
VA12	0.683			
VA3	0.655			
VA16	0.653			
VA10	0.613	0.465		
VA5	0.610			
VB6		0.822		48.055%
VB1		0.775		
VB3		0.758		
VB5		0.690		
VB4		0.633		
VB2		0.627		
VC5			0.838	63.534%
VC4			0.779	
VC1			0.776	
VC2			0.710	
VC6			0.699	
VC3			0.666	

The dimension of professional awareness is VA1–VA17; the dimension of professional conduct is VB1-VB6; the dimension of professional skills is VC1–VC6.

The factor loadings of all items for the three common factors were above 0.4. However, item VA10 had factor loadings over 0.4 on both the “Professional Skills” and “Professional Awareness” dimensions. This item was more relevant to the preset dimension, and the difference in the loading values was significant. The sample size may not be sufficient to provide high discriminability in the factor structure. Consulting experts and considering the actual situation, increasing the sample size is likely to reduce this type of error and widen the gap in factor-loading values; thus, this item was retained.

#### Confirmatory factor analysis

##### Step 1: confirmatory factor analysis of the primary measurement model of professionalism level

**Figure 1:**
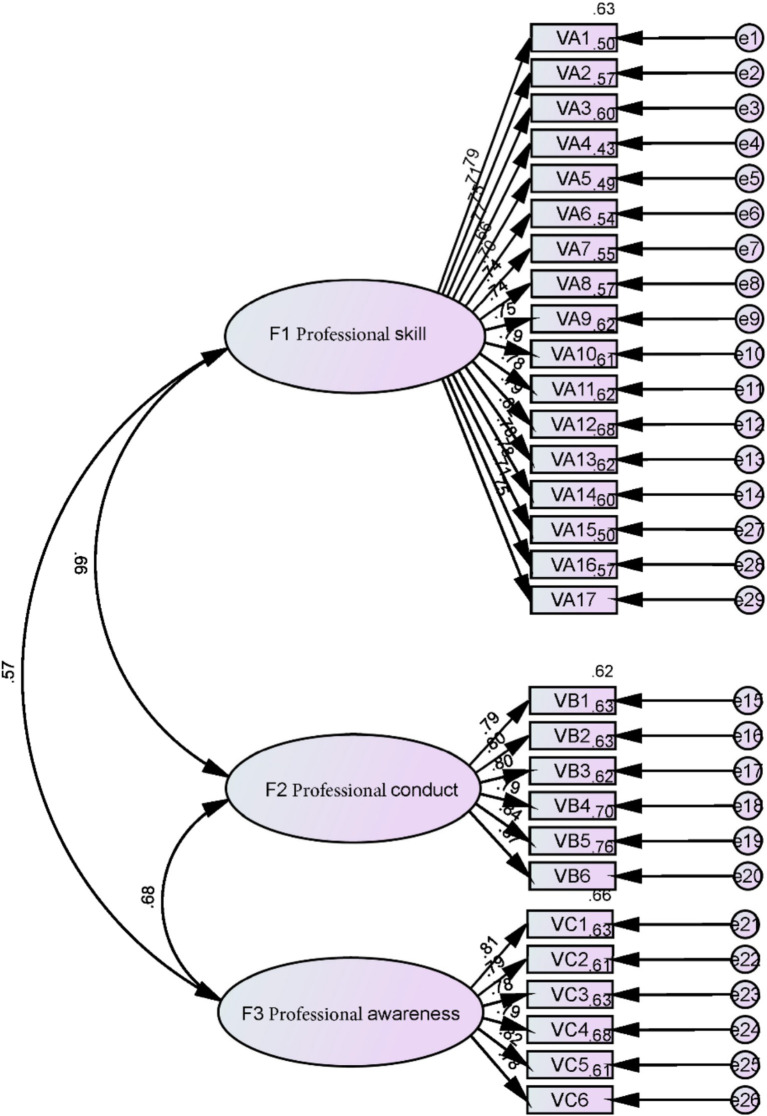
First-order three-factor correlated measurement model diagram (A0).

Drawing from foundational research and theoretical frameworks from earlier studies, an initial measurement model for Village doctors’ professionalism was devised. This first-order three-factor correlated model (A_0_) categorizes the factors derived from exploratory factor analysis into three interrelated second-order latent variables. The 29 indicators under each factor were used to assess validity, and a model diagram was constructed (Figure 1).

##### Step 2: model parameter estimation value inspection and analysis

Table 4 presents the model parameter estimates. The standardized factor loadings for each observed variable on the latent variable in the model ranged from 0.658 to 0.869, with none exceeding 0.95. The standard errors were positive and relatively small. The CR (*t*-value) is greater than 2.58 for all, indicating significance at the 0.01 level, and the *p*-values also pass the significance tests. Therefore, there were no estimation violations in the model, suggesting that the measurement items in this study were appropriately set and could reflect the professionalization level of village doctors from different perspectives.

**Table 4:** A_0_ results of model parameter estimates.

Variables	Indicator	Standardized loadings	Factor loading	S.E.	CR(t-value)	*P*	CR	AVE
F_1_	VA1	0.795	1.000	0.021	12.427	<0.001	0.966	0.591
	VA2	0.708	0.878	0.027	12.820	<0.001		
	VA3	0.755	0.944	0.024	12.644	<0.001		
	VA4	0.773	1.306	0.022	12.554	<0.001		
	VA5	0.658	0.740	0.031	12.954	<0.001		
	VA6	0.699	1.054	0.028	12.848	<0.001		
	VA7	0.736	0.820	0.025	12.723	<0.001		
	VA8	0.743	0.922	0.025	12.696	<0.001		
	VA9	0.752	0.883	0.024	12.655	<0.001		
	VA10	0.788	0.950	0.021	12.468	<0.001		
	VA11	0.778	0.923	0.022	12.525	<0.001		
	VA12	0.788	0.880	0.021	12.469	<0.001		
	VA13	0.824	1.008	0.018	12.202	<0.001		
	VA14	0.784	1.026	0.021	12.490	<0.001		
	VA15	0.777	0.927	0.022	12.532	<0.001		
	VA16	0.706	0.782	0.028	12.826	<0.001		
	VA17	0.754	0.964	0.024	12.649	<0.001		
F_2_	VB1	0.786	1.000	0.022	11.741	<0.001	0.921	0.661
	VB2	0.795	0.993	0.022	11.637	<0.001		
	VB3	0.795	0.963	0.022	11.636	<0.001		
	VB4	0.790	0.959	0.022	11.700	<0.001		
	VB5	0.839	1.092	0.018	10.962	<0.001		
	VB6	0.869	1.421	0.016	10.230	<0.001		
F_3_	VC1	0.810	1.000	0.021	11.122	<0.001	0.913	0.637
	VC2	0.793	0.997	0.022	11.370	<0.001		
	VC3	0.781	0.972	0.023	11.527	<0.001		
	VC4	0.795	0.970	0.022	11.345	<0.001		
	VC5	0.824	1.064	0.020	10.865	<0.001		
	VC6	0.783	0.958	0.023	11.501	<0.001		

The dimension of professional awareness is VA1–VA17; the dimension of professional conduct is VB1–VB6; the dimension of professional skills is VC1–VC6.

##### Step 3: Model fit evaluation

The results of the model fit indices used in this study are presented in Table 5. The absolute fit indices included an RMSEA value of 0.029, and both GFI and AGFI values were above 0.9. The incremental fit indices, including IFI, TLI, and CFI, were all greater than 0.9. The parsimonious fit index $\chi^{2}/df$ is less than 5. The fitting outcomes suggest that the hypothetical model established in this study has an excellent overall model fit and that the observed variables for the three latent variables can effectively represent their respective constructs.

**Table 5:** A_0_ results of model fit indices.

Fit indices	Recommended values	Results
$\chi^{2}/df$	<5	1.294
RMSEA	<0.08	0.029
GFI	>0.9	0.918
AGFI	>0.9	0.904
NFI	>0.9	0.937
IFI	>0.9	0.985
CFI	>0.9	0.985
SRMR	<0.08	0.030
TLI (NNFI)	>0.9	0.983

##### Step 4: construct validity assessment

① Convergent validity. All observed variables had standardized factor loadings within these limits; CR values ranged from 0.913 to 0.966, all above 0.6, and AVE values were all above 0.5. Hence, the hypothetical model is considered to have good convergent validity.

② Discriminant validity. Table 6 reveals that all standardized correlation coefficients between latent variables were below the square root of their respective AVE values, confirming good discriminant validity among the latent variables.

**Table 6:** Results of model discriminant validity.

	F_1_	F_2_	F_3_
F_1_	0.591		
F_2_	0.662***	0.661	
F_3_	0.574***	0.677***	0.637
Average square root	0.769	0.813	0.798

The data on the diagonal are the AVE values of the latent variables, and the rest of the data are the square roots of the completely standardized correlation coefficients between the latent variables, with ***p < 0.001.

#### Common method bias test

As shown in Table 7, RMSEA changes were below 0.05, while variations in CFI, GFI, AGFI, and TLI were less than 0.1. These minimal differences suggest that no substantial common method bias was present in the study.

**Table 7:** Controlling for unmeasured potential method test results.

Model	RMSEA	GFI	AGFI	CFI	SRMR	TLI	*Δ*RMSEA	*Δ*GFI	*Δ*AGFI	*Δ*TLI
A_0_	0.029	0.918	0.904	0.985	0.03	0.983	0.015	−0.017	−0.014	−0.013
A_1_	0.014	0.935	0.918	0.997	0.02	0.996				

￼ derived from the comparison between the A0 model and A1 model.

### Propensity score matching results

#### The village doctor policy compares the professionalization levels of village doctors

Table 8 illustrates the disparities between the groups that implemented the policy and those that did not. A t-test analysis revealed statistically significant differences, particularly in the level of professionalization among village doctors (*p* < 0.001).

**Table 8:** Results of independent samples *t*-test.

	Treatment (*N* = 194)	Control (*N* = 165)	*t*	*p*	Mean difference
Overall score for professionalization level	119.423 ± 17.661	110.008 ± 17.801	5.012	<0.001	9.415
Professional skill	3.679 ± 0.626	3.352 ± 0.625	4.931	<0.001	0.327
Professional conduct	4.028 ± 0.723	3.783 ± 0.763	3.102	<0.01	0.245
Professional awareness	4.168 ± 0.641	3.865 ± 0.719	4.191	<0.0001	0.304

#### The relationship between the village doctor policy, and the professionalization level of village doctors

The Ordinary Least Squares (OLS) model was employed by scientists to evaluate the effects of the Village Doctor Policy on the professionalization of village doctors (Table 9). The findings revealed a substantial positive impact on overall professionalism (*p* < 0.001). Further subdividing into three dimensions of professionalization, the policy has statistically significant effects on professional skills ( β=7.02 , *p* < 0.01), professional conduct ( β=5.89 , *p* < 0.05), and professional awareness ( β=6.34 , *p* < 0.05). Among the control variables, the study finds that gender has a significant negative impact on professional conduct ( β=−0.193 , *p* < 0.05), suggesting that female village doctors display higher levels of occupational awareness compared to their male counterparts. Years of work experience ( β=0.23 , *p* < 0.05) and professional background ( β=4.12 , *p* < 0.01) have a significant positive effect on professional skills.

**Table 9:** OLS model results.

Variables	Professionalization level	Professional skill	Professional conduct	Professional awareness
Village doctor policy	8.401*** (<0.001)	0.310*** (<0.001)	0.194** (<0.001)	0.244*** (<0.001)
Gender	−3.361 (0.119)	−0.0808 (0.286)	−0.193** (<0.05)	−0.0928 (0.252)
Age	−0.084 (0.587)	−0.00513 (0.332)	0.000618 (0.935)	0.000601 (0.933)
Education	0.977 (0.558)	−0.0108 (0.853)	0.105 (0.129)	0.0732 (0.291)
Annual income	−0.744 (0.393)	−0.0402 (0.185)	−0.00537 (0.878)	0.00276 (0.935)
Years of work experience	0.290** (<0.05)	0.0115** (<0.05)	0.00860 (0.200)	0.00361 (0.573)
Daily work hours	7.198*** (<0.001)	0.223*** (<0.001)	0.240*** (<0.001)	0.252*** (<0.001)
Social security	−3.678 (0.357)	−0.177 (0.191)	−0.0438 (0.816)	−0.0297 (0.856)
Academic background	5.195* (<0.1)	0.191* (<0.1)	0.203 (0.154)	0.0573 (0.632)
Technical professional rank	2.933** (<0.05)	0.116** (<0.05)	0.0475 (0.383)	0.0845* (0.091)
Population	0.000463** (<0.05)	0.0000282*** (<0.05)	0.00000688 (0.445)	−0.0000154* (<0.1)
Professional license	13.41*** (<0.001)	0.376** (<0.05)	0.560*** (<0.05)	0.452** (<0.05)
_cons	63.38*** (<0.001)	2.166*** (<0.001)	1.762*** (<0.001)	2.009*** (<0.001)
N	359			

*p < 0.1, **p < 0.05, ***p < 0.001.

#### Selection bias: a further analysis based on the PSM method

**Figure 2:**
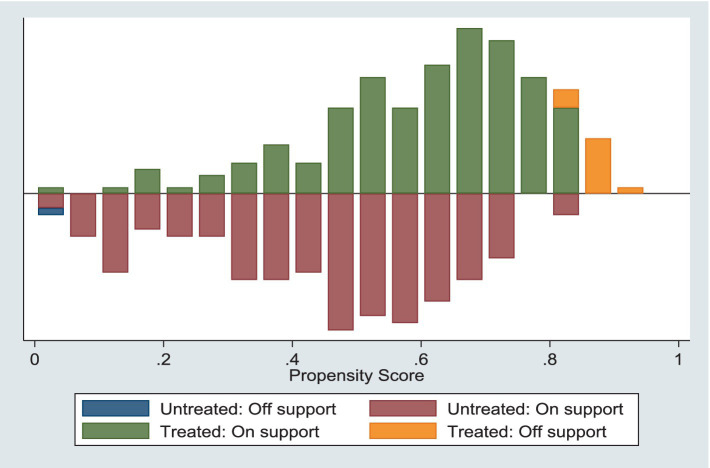
Samples collectively support domain testing.

**Figure 3:**
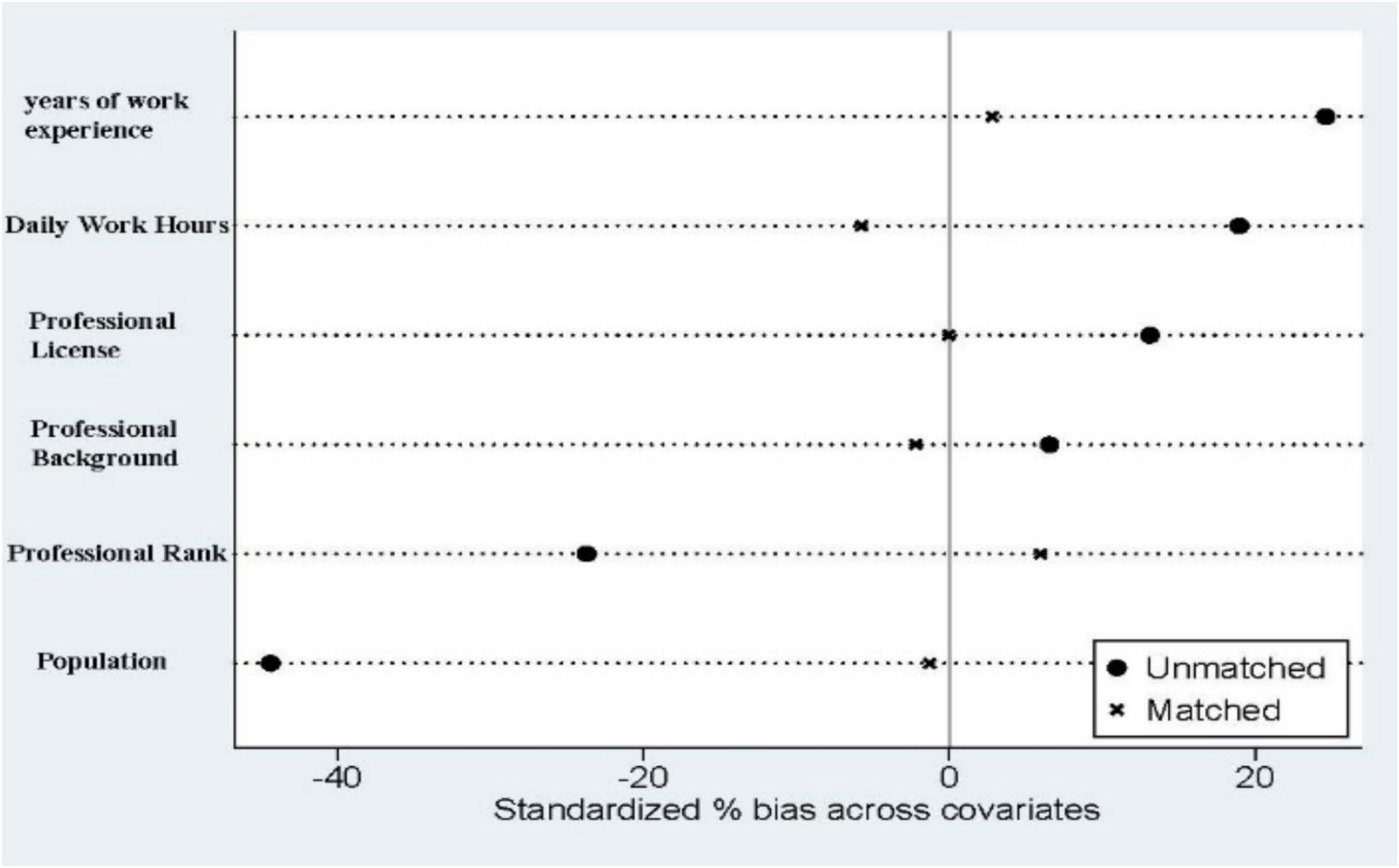
Comparison of standard deviations of control variables before and after matching.

Figure 2 shows an overlap in the propensity scores for village doctors in both policy areas, with most sample values being within common ranges. Figure 3 indicates that the standard deviation of each control variable decreases to less than 20% after matching. Table 10 reveals no statistically significant differences in the control variables post-matching, suggesting that selectivity bias due to sample self-selection was significantly reduced and that the samples met the conditional independence assumption. Thus, the data passed the equilibrium and common support domain tests, indicating an effective sample-matching outcome and validating the use of the propensity score matching method for further estimation. This confirmed that the sample-matching effect was relatively stable.

**Table 10:** Balance test for control variables.

Variable	Unmatched matched	Treated	Control	%bias	%reduct |bias|	*p* > |t|
Years of work experience	Unmatched	21.144	18.352	24.6	88.5	0.022
	Matched	19.934	19.613	2.8		0.778
Daily work hours	Unmatched	4.294	4.133	19.0	69.9	0.073
	Matched	4.243	4.291	−5.7		0.580
Professional background	Unmatched	0.773	0.061	6.6	66.9	0.537
	Matched	0.066	0.072	2.2		0.836
Technical professional rank	Unmatched	1.516	1.685	−23.7	74.7	0.025
	Matched	1.525	1.482	6.0		0.547
Population	Unmatched	1264.4	2418.8	−44.4	97.2	<0.001
	Matched	1317.1	1349.9	−1.3		0.766
Professional license	Unmatched	0.985	0.964	13.1	100.0	0.208
	Matched	0.983	0.983	0.0		1.000

#### The propensity score matches the estimated results

Table 11 reveals that the Village Doctor Policy significantly affects village doctors’ professionalism, showing a clear disparity between those who implemented the policy and those who did not. Table 11 details the Propensity Score Matching (PSM) results, highlighting the policy’s impact on professionalization. The ATT coefficient is 6.536 for k-Nearest neighbors matching, 9.017 for caliper matching, and 6.110 for kernel matching. These results indicate that the policy enhanced the professionalism of village doctors. We also matched propensity scores for the dimensions of professionalism level. The results of the three matching methods show that policy has a significant positive effect on professional skills. The highest mean difference in professional skills was found under the caliper matching method (difference = 0.315, *p* < 0.01). In terms of professional conduct and professional awareness, the direction of the policy’s impact is generally positive but slightly less significant, reaching a marginally significant level only under some of the methods. Notably, the increase in professional conduct (difference = 0.232, *p* < 0.05) and professional awareness (difference = 0.286, *p* < 0.05) under the caliper matching method was statistically significant.

**Table 11:** The propensity score matches the estimated results.

Dependent variable	Independent variable					
	Village doctor policy					
	Matching types	Treated	Controlled	Difference	S.E.	*t*
Professionalization level	k-Nearest neighbors (*k* = 4)	118.799	112.263	6.536	2.688	2.43**
	Caliper (Cal = 0.02)	118.581	109.563	9.017	3.325	2.71***
	Kernel	118.799	112.689	6.110	2.454	2.49**
Professional skill	k-Nearest neighbors (*k* = 4)	3.658	3.402	0.255	0.094	2.72***
	Caliper (Cal = 0.02)	3.653	3.341	0.315	0.114	2.76***
	Kernel	3.658	3.420	0.238	0.086	2.76***
Professional conduct	k-Nearest neighbors (*k* = 4)	4.003	3.856	0.147	0.115	1.28
	Caliper (Cal = 0.02)	3.991	3.759	0.232	0.135	1.72*
	Kernel	4.003	3.876	0.127	0.104	1.22
Professional awareness	k-Nearest neighbors (*k* = 4)	4.157	4.004	0.152	0.105	1.46
	Caliper (Cal = 0.02)	4.140	3.853	0.286	0.127	2.26**
	Kernel	4.157	4.000	0.157	0.096	1.63

*p < 0.1, **p <0.05, ***p < 0.01.

## Discussion

This study assessed the impact of village doctors’ recruitment policies on the aspect of professionalism enhancement of village doctors. The results of the study show that the policy measures have made significant progress in promoting the professionalization of village doctors, particularly in the area of professional skills upgrading. One of the key changes is the transfer of recruitment authority from village committees to township health centers, an adjustment that not only provides government endorsement for recruitment, but also attracts more medical students to join the profession due to the enhanced treatment guarantee for village doctors. At the same time, the integration of village doctors into the management system of township health centers has also raised the professional requirements for the original staff, leading to an improvement in overall professional skills and medical knowledge. In addition, the management system enables village doctors to systematically receive training organized by the township health hospitals, which effectively promotes skill enhancement and brings their competence more in line with the requirements of institutional norms (26, 27). On the other hand, the policy strengthened the integration of resources and administrative support between rural doctors and township health centers, alleviating the persistent infrastructure shortage (28). As a result, the policy has had a significant impact on improving the professional skills of village doctors.

Consistent with the findings of existing studies (22), this policy has a positive impact on the professional conduct and professional awareness of village doctors. After the implementation of the policy of ‘village recruitment and village employment’, township health hospitals will be responsible for the management of the service norms of all village doctors in the village health offices under their jurisdiction (29), and at the same time, village doctors will be changed to formal professional status, which is conducive to improving the professional conduct and awareness of the village doctor team. However, the effect on improving professional conduct and awareness is not as effective as the improvement of professional skills, probably because “Township-Hired Village Doctors” focuses more on assessing and motivating the professional skills of village doctors.

The results of the study showed that years of working experience, educational background, license certificates and titles and number of populations served had a significant effect on professional skills. Factors such as years of working experience and academic background reflect the ability of village doctors to accumulate experience and professional knowledge reserves and thus their professional skills. Whereas some of the studies showed that primary care doctors with age their professional conduct also grows (30). In this study, age had no significant effect on the level of professionalism of village doctors, which may be due to the general enhancement of the policy of village employment on the professional conduct of the village doctor workforce. Feng Wan et al. showed no significant difference in gender (30), and in this study gender had a significant effect on the professional conduct of village doctors, perhaps women pay more attention to details and standardized operations.

## Strengths and limitations

Previous studies have mostly emphasized the impact of rural doctors’ job satisfaction, professional identity, educational background and training on rural doctors’ retention and recruitment (22, 25, 31). For the career development of rural doctors, studies have explored the dilemmas faced by rural doctors’ career development from the perspective of career-embedded involution or sectionalism (29, 32). Research on the policy of rural-village employment mainly focuses on the dilemmas of policy implementation. This study is the first to empirically analyze the impact of the rural employment policy on the career development of rural doctors, and to provide a reference for the construction of the rural doctor team.

Nevertheless, the study still has some limitations. This study employed a cross-sectional design, capturing the professionalization level of village doctors as a dynamic process but did not account for its temporal aspects. Future research should use longitudinal cohort studies to examine the impact of professionalization at various stages under different policy conditions. Although we modified and tested the questionnaire for validity and reliability based on existing surveys, the limited sample size may constrain the generalizability of the findings to the broader professional context of village doctors in China.

Although the propensity score matching (PSM) method controls selection bias to a certain extent, it only adjusts for observable variables and cannot effectively deal with potential unobserved confounders, which may still lead to residual bias, thus limiting the robustness of causal interpretation. To further enhance the rigor of the study, subsequent studies may attempt to conduct sensitivity analyses to test the potential impact of unobserved confounding variables to enhance the credibility of causal inferences.

## Conclusion

This study highlights the positive impact of the Village Doctor Policy on the professionalization of village doctors. The findings suggest that the policy of “Township-Hired Village Doctors” has had a significant positive impact on the career development of village doctors in China, significantly improving their professional skills, professional awareness and professional conduct. These improvements suggest that policy interventions and healthcare reforms have had a positive impact on the working conditions and career trajectories of village doctors. To sum up, the policy has made positive progress in upgrading the professionalism of the rural doctor workforce, but there is still a need to optimize the implementation of the policy. However, challenges remain, including workload pressures and the need for continuous professional development.

It is recommended that fiscal support be expanded through the establishment of a dedicated village healthcare fund and public–private partnership models, which would elevate village doctors’ remuneration and modernize infrastructure. Moreover, equipping village health facilities with modern devices (e.g., portable ultrasound machines and telemedicine tools) and optimizing the pharmaceutical supply chain are crucial for ensuring adequate material resources in all remote areas, complemented by transportation subsidies. Additional measures include recruiting auxiliary personnel (such as nurses) and arranging for expert visits to village areas to reinforce human resources, thereby alleviating the workload of village doctors and enhancing the support network. Integrating township health centers through standardized salary and benefits, as well as unified electronic health record systems, will ensure that village doctors enjoy equal protections and data interoperability. National guidelines clarifying the roles of village doctors and promoting their social recognition are also warranted. Particularly important is the implementation of performance-based incentives—such as bonuses tied to improved patient health outcomes or satisfaction—regular training in areas like geriatric care and chronic disease management, and opportunities for career advancement (e.g., serving as leaders of village health projects).

Collectively, these multidimensional support measures would consolidate the policy’s achievements, address resource disparities and constraints in professional development, and transform village doctors into the backbone of village healthcare, thereby advancing China’s goals of health equity and universal coverage. These strengthened policy measures not only build on improvements in professional identity and job satisfaction but also address challenges such as an aging workforce and regional disparities. By adopting a more comprehensive and proactive approach, the Village Doctor Policy can serve as a model for sustainable rural healthcare reform, ensuring equitable access to high-quality medical services across China’s vast rural regions.

## Data Availability

The original contributions presented in the study are included in the article/supplementary material, further inquiries can be directed to the corresponding author.
